# Malassezia responds to environmental pH signals through the conserved Rim/Pal pathway

**DOI:** 10.1128/mbio.02060-24

**Published:** 2024-08-27

**Authors:** Kaila M. Pianalto, Calla L. Telzrow, Hannah Brown Harding, Jacob T. Brooks, Joshua A. Granek, Eduardo Gushiken-Ibañez, Salomé LeibundGut-Landmann, Joseph Heitman, Giuseppe Ianiri, J. Andrew Alspaugh

**Affiliations:** 1 Department of Medicine, Duke University School of Medicine, Durham, North Carolina, USA; 2 Department of Molecular Genetics and Microbiology, Duke University School of Medicine, Durham, North Carolina, USA; 3 Department of Physics and Astronomy, University of North Carolina, Chapel Hill, North Carolina, USA; 4 Department of Biostatistics and Bioinformatics, Duke University School of Medicine, Durham, North Carolina, USA; 5 Section of Immunology at Vetsuisse Faculty, University of Zurich, Zurich, Switzerland; 6 Institute of Experimental Immunology, University of Zurich, Zurich, Switzerland; 7 Department of Pharmacology and Cancer Biology, Duke University School of Medicine, Durham, North Carolina, USA; 8 Department of Cell Biology, Duke University School of Medicine, Durham, North Carolina, USA; 9 Department of Agricultural, Environmental and Food Sciences, Università degli Studi del Molise, Campobasso, Italy; The University of Texas Health Science Center at Houston, Houston, Texas, USA

**Keywords:** *Malassezia sympodialis*, mycosis, alkaline response, fungal pathogenesis, fungal infection

## Abstract

During mammalian colonization and infection, microorganisms must be able to rapidly sense and adapt to changing environmental conditions including alterations in extracellular pH. The fungus-specific Rim/Pal signaling pathway is one process that supports microbial adaptation to alkaline pH. This cascading series of interacting proteins terminates in the proteolytic activation of the highly conserved Rim101/PacC protein, a transcription factor that mediates microbial responses that favor survival in neutral/alkaline pH growth conditions, including many mammalian tissues. We identified the putative Rim pathway proteins Rim101 and Rra1 in the human skin colonizing fungus *Malassezia sympodialis*. Gene deletion by transconjugation and homologous recombination revealed that Rim101 and Rra1 are required for *M. sympodialis* growth at higher pH. In addition, comparative transcriptional analysis of the mutant strains compared to wild-type suggested mechanisms for fungal adaptation to alkaline conditions. These pH-sensing signaling proteins are required for optimal growth in a murine model of atopic dermatitis, a pathological condition associated with increased skin pH. Together, these data elucidate both conserved and phylum-specific features of microbial adaptation to extracellular stresses.

**IMPORTANCE:**

The ability to adapt to host pH has been previously associated with microbial virulence in several pathogenic fungal species. Here we demonstrate that a fungal-specific alkaline response pathway is conserved in the human skin commensal fungus *Malassezia sympodialis* (*Ms*). This pathway is characterized by the pH-dependent activation of the Rim101/PacC transcription factor that controls cell surface adaptations to changing environmental conditions. By disrupting genes encoding two predicted components of this pathway, we demonstrated that the Rim/Pal pathway is conserved in this fungal species as a facilitator of alkaline pH growth. Moreover, targeted gene mutation and comparative transcriptional analysis support the role of the *Ms* Rra1 protein as a cell surface pH sensor conserved within the basidiomycete fungi, a group including plant and human pathogens. Using an animal model of atopic dermatitis, we demonstrate the importance of *Ms* Rim/Pal signaling in this common inflammatory condition characterized by increased skin pH.

## INTRODUCTION

Changes in temperature, nutrient availability, or environmental pH create stressful conditions for microorganisms requiring continuous cellular adaptation for survival. In the case of pathogenic organisms, the shift from the ambient environment to the human host results in changes in many of these conditions, in addition to exposure to the host immune system. Similarly, commensal microorganisms must adapt to the unique environmental stresses presented by their specific niches within the host.

Extracellular pH can vary widely as microbes move from the environment to the human host. Even within the human body, pH can vary from the acidic pH of the stomach to the more neutral pH of blood, to the basic pH of bile. Human skin tends to be more acidic than blood or other body sites, with pH levels that can vary from pH 4 to pH 6 on healthy adult skin (1). This is an ideal pH for optimal growth of many fungi including *Malassezia sympodialis* (*Ms*) (2), a skin commensal microbe and opportunistic pathogen. However, in inflammatory skin conditions such as atopic dermatitis, the skin pH increases, representing an environmental trigger for this yeast-like species (2). In addition, other *Malassezia* species are adapted for colonization of the human gut. Recent reports reported that *Malassezia* can migrate from the more acidic upper gastrointestinal tract to more alkaline micro-niches in the pancreas, demonstrating how certain fungal species can adapt to fluctuations of pH within a mammalian host (3).

The fungus-specific Rim/Pal pathway includes a conserved cascading series of interacting proteins that sense and respond to changes in pH (4–6). First described in model ascomycete fungi such as *Saccharomyces cerevisiae* and *Aspergillus nidulans*, this signaling pathway initiates a response to pH changes at the cell surface through the Rim21/PalH pH sensor (7–9). This signal is then propagated through a conserved signaling cascade, with eventual cleavage and activation of the Rim101/PacC zinc-finger transcription factor (10–12). Once activated, this transcription factor translocates to the nucleus where it regulates the expression of many genes, resulting in an adaptive cellular response to alkaline pH stress (6, 10, 13, 14). By convention, this pathway has been referred to as the Pal pathway in filamentous fungi, and as the Rim pathway in fungi that grow predominantly as yeasts, such as *Saccharomyces cerevisiae*, *C. albicans*, and *C. neoformans*. Studies of this pathway in pathogenic fungi have revealed that many virulence factors require Rim/Pal pathway activation to be expressed. For example, in *Candida albicans*, the Rim pathway is required for the yeast-hyphal transition that is necessary for tissue invasion (15, 16). In addition, in the basidiomycete fungus *Cryptococcus neoformans*, activation of the Rim pathway is required for full expression of many virulence-associated phenotypes, including induction of the polysaccharide capsule and the formation of titan cells (10, 14, 17, 18).

Many Rim/Pal pathway proteins are conserved between the ascomycete fungi and the basidiomycete fungi *Cryptococcus neoformans* and *Ustilago maydis*, such as the components of the proteolysis complex (Rim20/PalA, Rim23/PalC, and the Rim13/PalB protease) and the Rim9/PalI chaperone (18–20). Homologs of more upstream components comprising the pH-sensing complex, including the Rim21/PalH pH sensor and the Rim8/PalF arrestin, are notably absent in the genomes of basidiomycetes (18, 19). The *C. neoformans* Rra1 protein was recently identified as a cell surface-associated protein that acts upstream of the Rim proteolysis complex to activate this pathway. While lacking sequence homology with the ascomycete Rim21 protein, *Cn* Rra1 shares functional and structural similarities with this established pH sensor (18). Importantly, while Rim21 homologs are absent from the genomes of many basidiomycete fungi, Rra1 homologs are readily apparent in sequenced basidiomycete genomes (18). We also identified a novel Rra1 interactor, Nucleosome Assembly Protein 1 (Nap1), which is required for activation of the Rim pathway in *C. neoformans*. However, Nap1 homologs do not appear to be similarly involved in the Rim pathway of *S. cerevisiae* (21). We therefore explored the degree of functional conservation of potential Rim pathway proteins in other basidiomycetes, such as *M. sympodialis*.

In this study, we established that the *M. sympodialis* Rim101 transcription factor and the putative Rra1 pH sensor are each required for survival at alkaline pH. In this way, we demonstrated that Rra1 is likely a conserved, basidiomycete-specific Rim pathway component. We also examined the transcriptional output of the *M. sympodialis* Rim pathway at alkaline pH, giving insight into the cellular processes involved in *M. sympodialis* survival in this stressful condition. Finally, we validated the relevance of the *Ms* Rim pathway in the interaction of this commensal fungus with the host by examining the innate immune response to fungal challenge *in vitro* and by evaluating the fitness of the fungus in the atopic skin environment *in vivo*.

## MATERIALS AND METHODS

### Strains, media, and growth conditions

The strains used in this study are listed in Table S1. Strains were routinely grown on modified Dixon’s (mDixon) medium [3.6% malt extract (Bacto), 1% mycological peptone (Oxoid), 1% ox bile (HiMedia), 1% vol/vol Tween 60 (Sigma), 0.4% glycerol; 2% Bacto agar added for plates] (22, 23). For the mDixon medium that was adjusted to specific pH, 150 mM HEPES was added to the medium as a buffering agent, and the pH was adjusted by the addition of either concentrated HCl or NaOH. For *in vivo* experiments, strains were grown in a differently modified Dixon medium containing 3.6% malt extract (Sigma), 2% ox bile (Sigma), 0.6% bacterial peptone (Oxoid), 1% Tween 40 (Sigma), 0.2% glycerol (Sigma), and 0.2% oleic acid (Sigma) (24). Strains were cultured at 30°C unless otherwise indicated. *Agrobacterium tumefaciens* cultures were maintained on YT agar or FB broth (YT agar: 0.8% Bacto tryptone, 0.5% Yeast Extract, 0.5% NaCl, 1.5% glucose; FB broth: 2.5% Bacto tryptone, 0.75% yeast extract, 0.1% glucose, 0.6% NaCl, 50 mM Tris-HCl, pH 7.6) at 30°C.

### *Agrobacterium tumefaciens*-mediated transformation

The *M. sympodialis RIM101* and *RRA1* genes were identified in the *M. sympodialis* genome assembly *via* BLASTp analysis (25). To generate *M. sympodialis mutants*, targeted deletion constructs consisting of 1.5 kb of genomic sequence upstream and downstream of the target genes and the *NAT* resistance marker were cloned into the T-DNA regions of plasmid pGI3 (26) using *in vivo* recombination in *Saccharomyces cerevisiae* as previously described (23). Primers used to create the deletion constructs are shown in Table S2. The resulting plasmid was transformed into *A. tumefaciens* EHA105 *via* electroporation and subsequent selection on YT agar supplemented with 50 µg/mL kanamycin.

*Agrobacterium*-mediated transformation was performed as described previously with some modifications (23, 27). Briefly, *M. sympodialis* ATCC 42132 was incubated for 2 days at 30°C in mDixon liquid medium, and *A. tumefaciens* strains containing pKP38 (*RIM101* deletion) or pKP39 (*RRA1* deletion) were incubated overnight in FB + kanamycin at 30°C. *A. tumefaciens* cultures were diluted to an OD_600_ of 1 in Induction Medium (IM) (335) and incubated a further 4 hours at 30°C. *M. sympodialis* cells and induced *A. tumefaciens* cells were mixed at a 5:1 *Malassezia:Agrobacterium* ratio and pelleted at 5,000 rpm, for 10 minutes. The resulting cell pellet was spotted onto nylon membranes on modified IM (mIM) plates [IM medium supplemented with 0.4% ox bile, 0.4% vol/vol Tween 60 (Sigma), 0.1% vol/vol Tween 20] and incubated at RT for 7 days. After 7 days, cells were scraped from the membranes into sterile H_2_O and pelleted at 3,000 rpm for 5 minutes (to preferentially pellet the *Malassezia* cells over the *Agrobacterium* cells). The resulting pellets were resuspended in sterile H_2_O and spread onto mDixon medium with cefotaxime + either nourseothricin or neomycin (300 µg/mL cefotaxime, 100 µg/mL nourseothricin, 200 µg/mL neomycin G418).

Resulting colonies were screened by PCR to confirm homologous recombination of the gene deletion constructs into the endogenous locus using primers listed in Table S3 (21). Independent mutant strains for each gene were made by separate, completely independent transformations. All tested phenotypes were concordant between independent mutant strains; therefore, results from single or both mutants for each gene are demonstrated in the Results.

### Quantitative reverse-transcriptase PCR

As a pilot experiment prior to RNA sequencing, *M. sympodialis RIM101* expression was measured *via* RT-qPCR, per previous methods (21). *M. sympodialis* was incubated overnight in mDixon at pH 4. Cells were pelleted by centrifugation and washed twice in sterile H_2_O, then resuspended in mDixon medium, mDixon buffered to described pH levels, RPMI +10% fetal bovine serum (FBS), or Dulbecco's modified Eagle medium (DMEM) +10% FBS. *M. sympodialis* was incubated in these media at 30°C for 90 minutes. At this time, *M. sympodialis* samples were pelleted by centrifugation for 5 minutes at 4,000 rpm, 4°C. Cells were washed 2× with cold sterile H_2_O, then flash-frozen on dry ice. RNA was purified using Trizol phenol-chloroform extraction (Invitrogen). cDNA was prepared using the AffinityScript cDNA synthesis kit (Agilent). qRT-PCR was performed using PowerUp SYBR Green (ThermoFisher). Relative *MsRIM101* gene expression levels compared to pH 4 conditions were calculated using the ΔΔC_T_ method with the *MsTUB1* tubulin gene as control (21). RT-PCR primers for the *MsRIM101* and *MsTUB1* genes are shown in Table S4.

### RNA sequencing

To prepare samples for RNA sequencing, *M. sympodialis* WT, *rim101*Δ, and *rra1*Δ strains were grown in biological triplicate for 18 hours in mDixon liquid medium at 30°C. Cultures were pelleted at 4,000 rpm for 10 min, washed 1× with sterile H_2_O, and resuspended at an OD_6oo_ of 2.5 in 20 mL of mDixon pH 4 (Rim pathway-inactivating condition), mDixon pH 7.5 (Rim pathway-activating condition), or DMEM supplemented with 10% FBS (“host-like” condition). Cultures were incubated at 30°C for 90 minutes with shaking. Cultures were harvested by centrifugation at 4,000 rpm for 10 minutes at 4°C, then washed once with cold sterile H_2_O. Cell pellets were flash-frozen in a dry ice-ethanol bath and lyophilized overnight. RNA was extracted from the lyophilized pellets *via* a Trizol-chloroform extraction (Invitrogen). The resulting RNA was treated with DNase I (New England Biolabs) for 30 minutes at 37°C as described, then re-purified using Trizol and chloroform.

RNA Sequencing was performed in collaboration with the Duke University Center for Genomic and Computational Biology Sequencing and Genomic Technology Shared Resource. An mRNA library was prepared using a Kapa Stranded mRNA-Seq library prep kit. Stranded mRNA-Seq was performed on an Illumina HiSeq 4000 with 50 bp single-end reads. Reads were mapped to the *M. sympodialis* ATCC 42132 reference genome (obtained from Ensembl, accessed March 2021) using STAR alignment software (28). Differential expression analyses were performed in R using an RNA‐Seq Bioconductor workflow (29), followed by the DESeq2 package (30). Genes were considered statistically differentially expressed if they had an adjusted *P* value [false-discovery rate (FDR)] of <0.05. Functional prediction for differentially regulated genes (±1 log2 fold change) was performed using Fungi DB (www.fungidb.org [Release 63]) (31) entering the MSYG gene identifier for each transcript of interest.

The test of correlation between fold-change values for *rra1*Δ and *rim101*Δ mutants was calculated using Kendall’s τ test, with the alternative hypothesis that fold-change values were positively correlated. We initially only considered genes with significantly differential expression (adjusted *P*-value <= 0.05) in both mutants relative to WT. Results for among genes with significant differences in expression, using Kendall’s τ, of gene expression in between the growth at pH 7.5, in DMEM, and at pH 4 were, respectively, τ = 0.8206107, *P*-value <2.2 × 10^-16^; τ = 0.71747, *P*-value <2.2 × 10^-16^; τ = 1, *P*-value = 0.1666667). Because there are only three genes that have significantly different expression at pH 4 (in both *rra1*Δ and *rim101*Δ mutants compared to WT), we repeated this calculation for all genes and again found significant results (with less dramatic τ values) for growth at pH 7.5 and in DMEM, but, again, not at pH 4 ((respectively, τ = 0.6022764, *P*-value <2.2 × 10^-16^; τ = 0.5515425, *P*-value < 2.2 × 10^-16^; τ = −0.0465178, *P*-value = 0.999999). This analysis used the R packages: readxl, dplyr, ggplot2, and patchwork.

### Fungal survival in macrophages

The ability of the fungal strains to survive in the presence of macrophages was assessed by co-culture as previously described, with some alterations to accommodate *M. sympodialis* growth requirements (32, 33). Approximately 10^5^ J774A.1 murine macrophages suspended in DMEM (Thermo Fisher Scientific) were added to individual wells of a 96-well plate and incubated overnight at 37°C with 5% CO_2_. Following adherence to the 96-well plate, J774A.1 murine macrophages were activated with 10 nM phorbol myristate acetate (PMA) in RPMI 1640 medium (Corning) supplemented with 20% FBS for 1 hour at 37°C with 5% CO_2_. Following macrophage activation, *M. sympodialis* strains [WT, *rim101*Δ mutant strains (KPY34 and KPY36), and *rra1*Δ mutant strains (KPY38 and KPY39)], which had been incubated for 48 hours in mDixon medium, were washed three times in sterile water, normalized to an OD of 0.3 in RPMI 1640 medium supplemented with 20% FBS, and added to the activated J774A.1 murine macrophages [5 × 10^6^ fungal cells per well (10:1 fungal cells:macrophages)]. Co-cultures of J774A.1 murine macrophage and fungal cells were incubated for 4 hours at 37°C with 5% CO_2_. Phagocytosed fungal cells were collected by washing individual wells of the 96-well plate vigorously with sterile water. Collected fungal cells were plated onto mDixon agar to assess the number of viable *M. sympodialis* cells by quantitative culture. The results are reported as the average percentage (± SEM) of recovered CFU, normalized to the WT strain, generated from at least three biological replicates. Statistical significance was determined using one-way analysis of variance (ANOVA) and the Tukey-Kramer test (GraphPad Software, San Diego, CA).

Supplementation with 20% FBS provided exogenous lipids to support *M. sympodialis* lipid auxotrophy in this assay. RPMI 1640 medium was utilized specifically in this experiment to limit the impact of alkaline pH on the survivability of the *rim101*Δ and *rra1*Δ mutant strains. We found that a 4-hour incubation in RPMI 1640 medium supplemented with 20% FBS without J774A.1 murine macrophages did not impact the survivability of the *rim101*Δ and the *rra1*Δ mutant strains compared to the WT strain. Attempts at longer incubations, such as 24 hours, failed to recover viable fungi from co-culture. As a result, we used 4-hour co-cultures to directly assess the ability of the tested fungal strains to interact with and survive in the presence of macrophages.

### Macrophage activation assays

Bone marrow cells were isolated from C57BL/6 mice (Jackson Laboratories) as previously described (34, 35). Briefly, femurs were isolated from CO_2_-euthanized mice, and each bone marrow space was flushed with cold phosphate-buffered saline (PBS). Red blood cells were lysed in 1× RBC lysis buffer, and the remaining bone marrow cells were resuspended in 1× DMEM with 1 U/mL penicillin/streptomycin (PenStrep). Adherent cells were differentiated in bone-marrow-derived macrophage (BMM) medium [1× DMEM, 10% fetal bovine serum (FBS; non-heat inactivated), 1 U/mL penicillin/streptomycin] with 3 ng/mL recombinant mouse GM-CSF (rGM-CSF; R&D Systems or BioLegend) at a concentration of 2.5 × 10^5^ cells/mL in 150 × 15 mm petri plates at 37°C with 5% CO_2_. The media was refreshed after 3 days and the cells were harvested on day 7 as previously described, likely resulting in a mixture of BMMs and dendritic cells (DCs) (35). These cells were counted by hemocytometer (with Trypan blue to differentiate between live and dead cells), plated in BMM medium in 96-well plates at a concentration of 5 × 10^4^ cells per well, and incubated at 37°C with 5% CO_2_ overnight prior to fungal co-culture experiments.

BMM co-cultures with wild-type *M. sympodialis*, *C. neoformans,* and *C. albicans* as well as *M. sympodialis rim101*∆ and *rra1*∆ mutant strains were performed as described previously (34, 35). *M. sympodialis* strains [WT (ATCC 42132), *rim101*∆ (KPY34), and *rra1*∆ (KPY36)] were incubated for 3 days in DMEM media supplemented with 10% FBS (non-heat inactivated) and 1 U/mL penicillin/streptomycin at 30°C. The *C. neoformans* H99 strain was incubated for 2 days in DMEM media supplemented with 10% FBS (non-heat inactivated) and 1 U/mL penicillin/streptomycin at 30°C. Prior to co-culturing with BMMs, these cultures were transferred to 37°C for 16 hours. WT *C. albicans* (SC5314) cells were incubated for 1 day in DMEM without serum at 30°C prior to co-culture to avoid premature filamentation. Following these incubations, fungal cells were washed twice with PBS, counted, and added to wells of a 96-well plate containing BMM (5 × 10^5^ BMM/well) at a concentration of 5 × 10^6^ fungal cells per well (10:1 fungal cells:BMMs). Co-cultures were incubated for the indicated amount of time (either 3 or 6 hours) at 37°C with 5% CO_2_. Supernatants were collected and stored at −80°C overnight. Secreted cytokines [tumor necrosis factor (TNF)] were quantified in supernatants by enzyme-linked immunosorbent assay (ELISA MAX:Deluxe Set Mouse TNF; BioLegend). Data are represented as the average TNF levels (pg/mL) for 4–5 biological replicates per group (34, 35).

### Scanning electron microscopy

Scanning electron microscopy (SEM) was used to visualize the interactions between *M. sympodialis* strains and J774A.1 murine macrophages. Co-cultures were performed as described above, with some alterations. Individual ethanol-sterilized polydopamine-coated coverslips (18 mm diameter) were placed into the wells of a 12-well plate. Approximately 5 × 10^6^ J774A.1 murine macrophages suspended in RPMI 1640 medium supplemented with 20% FBS and 10 nM PMA were added to each well and incubated for 1 hour at 37°C with 5% CO_2_. Following adherence and activation, 48-hour incubated *M. sympodialis* cultures [WT, *rim101*Δ mutant (KPY34), and *rra1*Δ mutant (KPY38)] were washed three times in sterile water, normalized to an OD of 0.3 in RPMI 1640 medium supplemented with 20% FBS, and added to wells (5 × 10^6^ fungal cells per well (1:1 fungal cells:macrophages). Co-cultures of J774A.1 murine macrophages and fungal cells were incubated for 1  hour at 37°C with 5% CO_2_ to capture potential interactions (such as phagocytosis) between macrophages and fungal cells.

Co-cultures were fixed with 2.5% glutaraldehyde for 1 hour at room temperature and were subsequently washed three times with 1× PBS. Samples were dehydrated by immersing the coverslips in ethanol (30% for 5 minutes, 50% for 5 minutes, 70% for 5 minutes, 95% for 10 minutes, and 100% for 10 minutes performed twice). Samples were then critical point dried with a Tousimis 931 critical point dryer (Rockville, Maryland) and coated with gold-palladium using a Cressington 108 sputter-coater (Watford, United Kingdom). Coverslips containing the prepared samples were mounted and imaged on a Hitachi S-4700 scanning electron microscope (Tokyo, Japan).

### Murine skin colonization assay

Mouse experiments in this study were performed in strict accordance with the guidelines of the Swiss Animals Protection Law and under protocols approved by the Veterinary Office of the Canton Zurich, Switzerland (license number 142/2021). All efforts were made to minimize suffering and ensure the highest ethical and humane standards according to the 3R principles (36). WT C57Bl/6JRj mice were purchased from Janvier Elevage (France). All experiments were conducted at the Laboratory Animal Science Center of the University of Zurich under specific pathogen-free conditions. AD-like conditions were induced in the murine ear skin according to Moosbrugger-Martiz et al. (37) and Ruchti et al. (38). Briefly, 1.125 nm of MC903 (calcipotriol hydrate, Sigma) diluted in pure ethanol was applied on the dorsal and ventral side of both ears for 5 consecutive days and again for 4 days after a resting period of 2 days. The pH in MC903-treated mouse skin is higher (pH 7–7.5) compared to that of control skin (pH 5.5) (39). *M. sympodialis* strains (WT, *rim101*Δ, and *rra1*Δ) were grown in mDixon for 2 days, washed twice in PBS, and resuspended in native olive oil. A suspension of 100 µL containing 1 × 10^7^ yeast cells was applied topically onto the dorsal side of both ears while mice were anesthetized (40). After infection, MC903 treatment was continued on day 2 p.i. (for the 4-day infection experiment) or daily from days 2 to 5 (for the 7-day infection experiment) on the ventral side of the ear only to avoid interference of the EtOH solvent with fungal viability (38). Ear thickness was continuously monitored using the Oditest S0247 0–5 mm measurement device (Kroeplin). For determining the fungal loads in the skin, the ear tissue was transferred in water supplemented with 0.05% Nonidet P40 (AxonLab), homogenized with a TissueLyzer (Qiagen) for 6 minutes at 25 Hz, plated on mDixon agar, and incubated at 30°C for 3 to 4 days for colony counting.

## RESULTS

### Mutants in *M. sympodialis* Rim pathway signaling are hypersensitive to elevated pH and salt concentrations

**Fig 1:**
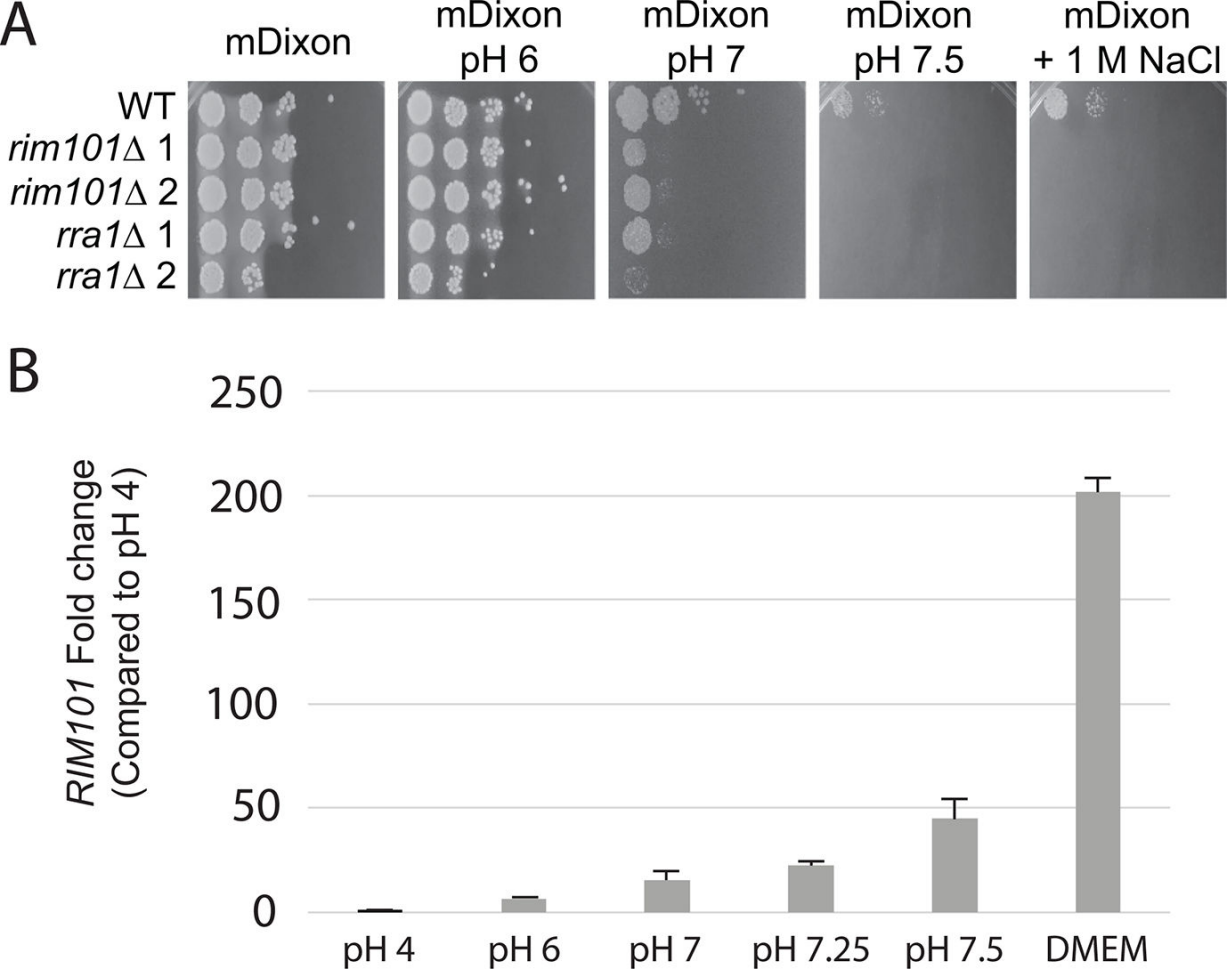
(**A**) *M. sympodialis RIM101* and *RRA1* genes are required for optimal growth at alkaline pH and in high salt conditions. Indicated strains were serially diluted and incubated in spot cultures on mDixon medium buffered to pH 6, pH 7, or pH 7.5, or mDixon medium supplemented with 1 M NaCl to examine growth phenotypes. Plates were imaged 6 days post-inoculation. (**B**) *M. sympodialis RIM101* gene expression in response to increasing pH and tissue culture medium. Wild-type *M. sympodialis* cells were incubated overnight in mDixon medium pH 4, then shifted to one of the following conditions for 90 minutes: mDixon buffered to pH 4, pH 6, pH 7, pH 7.25, or pH 7.5; or tissue culture medium (DMEM or RPMI +10% FBS). Relative transcript abundance of the *RIM101* gene was assessed by quantitative real-time PCR using the ΔΔC_T_ method and the *TUB2* tubulin gene as a control. Fold-change values for each condition were normalized to mDixon pH 4.

In other fungal species, the Rim/Pal pathway is involved in sensing and responding to increases in extracellular pH (4, 5, 14, 18). Accordingly, Rim pathway genes are required for survival at elevated pH as well as at elevated salt concentrations (15, 17, 41–43). To determine whether the *M. sympodialis* (*Ms*) Rim pathway is involved in similar cellular responses, two putative Rim pathway genes in the recently annotated *Ms* genome were identified based on sequence homology; MSYG_3336 encodes the closest homolog of the Rim101 transcription factor, and MSYG_4280 encodes the closest homolog of the Rra1 putative pH sensor. To explore their roles in the fungal response to extracellular stresses, two independent loss-of-function mutant strains were generated for each corresponding gene (Table S1). All tested phenotypes were concordant between the independent *Ms rra1*Δ mutants as well as between the independent *Ms rim101*Δ mutants. On media containing exogenous lipids, the wild-type *Ms* strain was able to grow well from a pH of 5 to a pH of 7.5; the *Ms rim101*Δ and *rra1*Δ mutant strains grew at rates similar to the WT at mildly acidic pH (pH 6) (Fig. 1A). However, they began to display growth defects at neutral pH 7, and growth was completely inhibited at pH 7.5 (Fig. 1A). Similar to Rim/Pal pathway mutants in other fungi, these *Ms* mutant strains displayed a growth impairment at high concentrations of NaCl (Fig. 1A). These results suggest that, as observed in other fungal genera, the *Ms* Rim101 transcription factor is involved in sensing and responding to alkaline pH and high salt conditions. Moreover, these data also suggest that Rra1 homologs play a conserved role in basidiomycete Rim/Pal pathways to sense and respond to alkaline pH signals.

### Comparative transcriptional analysis defines Rim pathway-regulated genes in *M. sympodialis*

Activation of the conserved and fungal-specific Rim signaling pathway results in the proteolytic cleavage of the Rim101 transcription factor, which then translocates to the nucleus to regulate transcriptional responses to alkaline pH, including induction of the expression of *RIM101* itself (14). To first determine pathway-activating signals, we performed quantitative real-time PCR to assess conditions associated with transcriptional activation of the *RIM101* gene. We performed this transcriptional analysis across a gradient of pH values in mDixon medium while also assessing *RIM101* transcript levels in tissue culture medium. *Ms RIM101* transcript levels increased in response to a more alkaline pH in a dose-dependent manner (Fig. 1B), similar to *RIM101/PacC* transcriptional induction observed in other fungal species (44, 45). In addition, *RIM101* is even more strongly transcriptionally induced in tissue culture media (DMEM and RPMI media, pH 7.4) than mDixon medium pH 7.5, indicating that signals in the tissue culture media in addition to pH are involved in activating the Rim pathway in this organism (Fig. 1B).

We performed deep RNA sequencing comparing the transcriptomes of the *Ms* WT, *rim101*Δ, and *rra1*Δ strains incubated for 90 minutes in mDixon pH 4, mDixon pH 7.5, and DMEM tissue culture medium (pH 7.4). Gene expression is highly correlated between *rra1*Δ and *rim101*Δ mutants, compared to WT, when grown at pH 7.5 or in DMEM, but there is little correlation between *rra1*Δ and *rim101*Δ mutants when they are grown at pH 4 (Fig. S1). This correlation is highly statistically significant at pH 7.5 or in DMEM (*P*-value < 2.2 × 10^-16^ for both), but there is no significant correlation for growth at pH 4 (*P*-value = 0.1666667).

Because there are only three genes that have significantly different expression at pH 4 in both *rra1*Δ and *rim101*Δ mutants compared to WT, we also calculated Kendall’s τ for all genes and again found significant results (also less dramatic) for growth at pH 7.5 and in DMEM, but not at pH 4 (respectively τ = 0.6022764, *P*-value < 2.2 × 10^-16^; τ = 0.5515425, *P*-value < 2.2 × 10^-16^; τ = −0.0465178, *P*-value = 0.999999)

**Fig 2:**
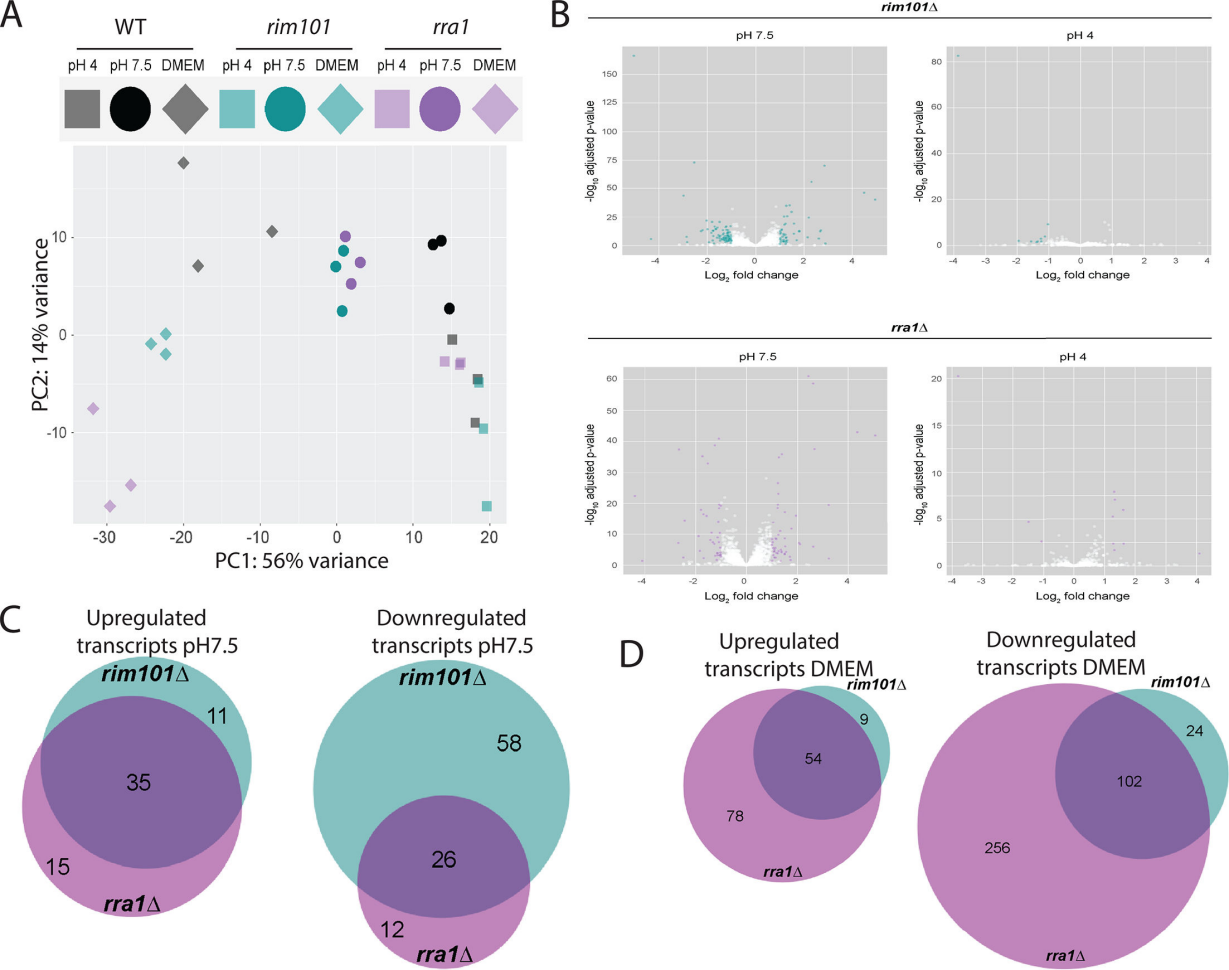
Comparative transcriptional analysis. The *M. sympodialis* WT, *rim101*Δ, and *rra1*Δ strains were incubated in each of the following conditions for 90 minutes prior to total RNA extraction for RNAseq analysis: mDixon medium pH 4, mDixon medium pH 7.5, DMEM. (**A**) Comparison of global transcriptome patterns by Multidimensional Scaling (MDS) analysis (three biological replicates for each indicated strain in each condition). (**B**) Volcano plots illustrating a number of genes with statistically significant alterations in transcript abundance at pH 7.5 and pH4. Genes with alterations in transcript abundance (±1 log_2_ fold change) are indicated in green (*rim101*Δ versus WT) or magenta (*rra1*Δ versus WT). (**C**) Venn diagram indicating the number of genes with statistically significant differences in transcript abundance (±1 log_2_ fold change) for indicated strains compared to WT at pH 7.5 (green = *rim101*Δ, magenta = *rra1*Δ). (**D**) Venn diagram indicating the number of genes with statistically significant differences in transcript abundance (±1 log_2_ fold change) for indicated strains compared to WT in DMEM (green = *rim101*Δ, magenta = *rra1*Δ).

We also analyzed the combined transcriptional data from each experimental sample using multidimensional scaling (MDS) analysis to visually compare variations in the transcriptomes of each data set. As expected, biological replicates of the same strain incubated at the same conditions tended to cluster more closely to each other than to other samples (Fig. 2A). This analysis indicates that overall patterns of transcriptional activity are very similar in mDixon medium at pH 4 among the WT, *rim101*Δ, and *rra1*Δ strains (Fig. 2A), consistent with work in other fungal species demonstrating specific activation of the Rim/Pal pathway in response to alkaline pH and other cell stress signals (4). By contrast, when these strains were incubated at elevated pH in mDixon medium buffered to pH 7.5 for 90 minutes, there were distinct patterns of transcriptional activity that distinguished the WT strain from the two mutant strains, which clustered together and clearly distinctly from the WT strain (Fig. 2A). A similar observation was made for samples obtained after growth in DMEM, although there was a higher variation among the *rim101*Δ and *rra1*Δ mutants. The relative transcriptional variation of individual genes between the WT and the *rim101*Δ/*rra1*Δ strains at each pH is demonstrated by Volcano plots (Fig. 2B).

At pH 4, there are very few genes differentially regulated between the WT and *rim101*Δ or *rra1*Δ mutant strains (Fig. 2B; Tables S5 and S6). However, at pH 7.5, 84 *Ms* genes displayed statistically significant decreased transcript abundance in the *rim101*Δ mutant strain compared to WT (< −1 log_2_ fold change), suggesting their transcriptional dependence on the putative Rim101 transcription factor (Fig. 2B and C; Tables S7 and S8). Of these genes, 26 (31%) demonstrated similar decreases in transcript abundance in the *rra1*Δ mutant (Fig. 2C; Tables S7 to S11). In addition, 46 genes displayed increased transcript abundance (> +1 log_2_ fold change) in the *rim101*Δ strain compared to WT, and 35 (76%) of these also had increased transcript abundance in the *rra1*Δ strain (Fig. 2B and C; Tables S7, S11 to S13).

Similar overlapping transcriptional patterns of the *Ms rim101*Δ and *rra1*Δ mutant strains are observed in DMEM tissue culture medium (pH 7.4): 126 *Ms* genes display decreased transcript abundance in the *rim101*Δ mutant strain compared to WT, and 102 (81%) of these genes also demonstrate decreased transcript abundance in the *rra1*Δ mutant; 63 genes have increased transcript abundance in the *rim101*Δ strain compared to WT, and 54 (86%) have similarly increased transcript abundance in the *rra1*Δ strain (Fig. 2D; Tables S14 to S20).

Assigning likely functions to specific genes in these data sets is limited by the incomplete annotation of the *M. sympodialis* genome (>20% of genes listed as encoding an “unspecified product”). However, we used FungiDB (31) to assist in assigning predicted functions for the proteins encoded by genes with altered transcription in either the *rim101*Δ or *rra1*Δ mutant strains at pH 7.5 and in DMEM (Tables S10, S12, S13, S16 to S19). We also manually defined functional categories of predicted function for genes with similar patterns of altered transcription in both the *rim101*Δ and *rra1*Δ strains in these two incubation conditions. A subset of these genes is listed in Tables 1 and 2. This analysis suggested potential *Ms* Rim pathway regulation at alkaline pH for multiple genes encoding proteins involved in membrane transport (MFS proteins; transporters of ammonium, amino acids, nucleosides, and ions) and intracellular trafficking (including the ESCRT II protein Vps25). A larger gene set displayed altered transcript abundance in DMEM between the *rim101*Δ/*rra1*Δ strains and WT. These genes similarly included those predicted to encode proteins involved in membrane transport and intracellular trafficking. Additional functional categories for genes with differential transcript abundance in DMEM in these Rim pathway mutants included cell cycle regulation, lipid metabolism, and cell surface modification. These categories are similar to those for Rim pathway-regulated genes in related basidiomycetes in which cell surface (cell wall and membrane) and cell cycle modifications are important components of the adaptive cellular response to environments with elevated pH (45).

**TABLE 1:** Functional categories enriched among the genes differentially expressed in DMEM in both the *rim101*∆ and *rra1*∆ mutant strains^*a*^

Functional category	Gene ID	Gene name	Predicted protein function
Upregulated			
Membrane transport	MSYG_3909	*ATO2*	Putative transmembrane protein involved in export of ammonia
	MSYG_2629		MFS domain-containing protein
	MSYG_3908		MFS domain-containing protein
	MSYG_3150	*FCY21*	Putative purine-cytosine permease
	MSYG_0038		MFS domain-containing protein
	MSYG_4234	*FCY2*	Purine-cytosine permease
	MSYG_0981		MFS domain-containing protein
	MSYG_4142	*SPA2*	Component of the polarisome
	MSYG_3302		MFS domain-containing protein
Cell cycle regulation	MSYG_1748		Csm1 domain-containing protein
	MSYG_1957	*PXL1*	Protein that localizes to sites of polarized growth
	MSYG_0194		Cyclin N-terminal domain-containing protein
	MSYG_0335		SH3 domain-containing protein
	MSYG_2712		Cyclin N-terminal domain-containing protein
Cell surface modifications	MSYG_4357		Chitin deacetylase
	MSYG_1041		Glyco *trans* 2-like domain-containing protein
Downregulated			
Protein synthesis	MSYG_1555		Prolyl-tRNA synthetase
	MSYG_0269		tRNA-dihydrouridine synthase
	MSYG_4576		tRNA-Glu
	MSYG_4544		tRNA-Glu
Cell cycle regulation	MSYG_0270		Spc7 domain-containing protein
	MSYG_1674		SRR1 domain-containing protein
	MSYG_1379		CRAL-TRIO domain-containing protein
Cell surface modifications	MSYG_0176		Mannose-P-dolichol utilization defect 1 protein homolog
	MSYG_1904	*ROT1*	Protein chaperone
	MSYG_1333		Alpha-1,2-Mannosidase
Intracellular trafficking	MSYG_3869		Vps8 domain-containing protein
	MSYG_2318		Adaptin N domain-containing protein
	MSYG_2610	*TRS33*	Core component of transport protein particle (TRAPP) complex
Lipid metabolism	MSYG_1989		Fatty acid hydroxylase domain-containing protein
	MSYG_0629	*ERG5*	C-22 sterol desaturase

^*a*^ Functional category assignment was performed manually based on predicted gene function using FungiDB annotations (2022).

**TABLE 2:** Functional categories enriched among the genes differentially expressed in the presence of alkaline pH (pH 7.5) in both the *rim101*∆ and *rra1*∆ mutant strains^*a*^

Functional category	Gene ID	Gene name	Predicted protein function
Upregulated			
Membrane transport	MSYG_0981		MFS domain-containing protein
	MSYG_3302		MFS domain-containing protein
	MSYG_0839		AA permease domain-containing protein
	MSYG_2629		MFS domain-containing protein
	MSYG_3150	*FCY1*	Putative purine-cytosine permease
Redox metabolism	MSYG_3153		L-ornithine N(5)-monooxygenase
	MSYG_4146		NMO domain-containing protein
	MSYG_4103	*CYB2*	Cytochrome b2 (L-lactate cytochrome-c oxidoreductase)
	MSYG_0029		Aldedh domain-containing protein
Intracellular transport	MSYG_0982	*YPT31*	Rab family GTPase
	MSYG_3478		CRAL-TRIO domain-containing protein
	MSYG_1774	*VPS25*	Component of the ESCRT-II complex
Fatty acid metabolism	MSYG_1824		SCP2 domain-containing protein
	MSYG_3126		Lipase 3 domain-containing protein
Downregulated			
Membrane transport	MSYG_1125		Ammonium transporter
	MSYG_1634	*MUP1*	Cation ATPase N domain-containing protein
	MSYG_3059		High-affinity methionine permease
	MSYG_3009		Na H Exchanger domain-containing protein
	MSYG_3517		MFS domain-containing protein
Redox metabolism	MSYG_1484		GMC OxRdtase N domain-containing protein

^*a*^ Functional category assignment was performed manually based on predicted gene function using FungiDB annotations (2022).

The *Ms RRA1* gene displayed transcriptional dependence on the Rim101 transcription factor in DMEM (−0.95 log_2_ fold change). This suggests that potentially biologically relevant changes in the transcript abundance of Rim101-regulated genes might be missed by arbitrary fold-change cut-offs and subsequent data set limitations.

### *M. sympodialis* interacts with macrophages *in vitro* in a Rim pathway-independent manner

**Fig 3:**
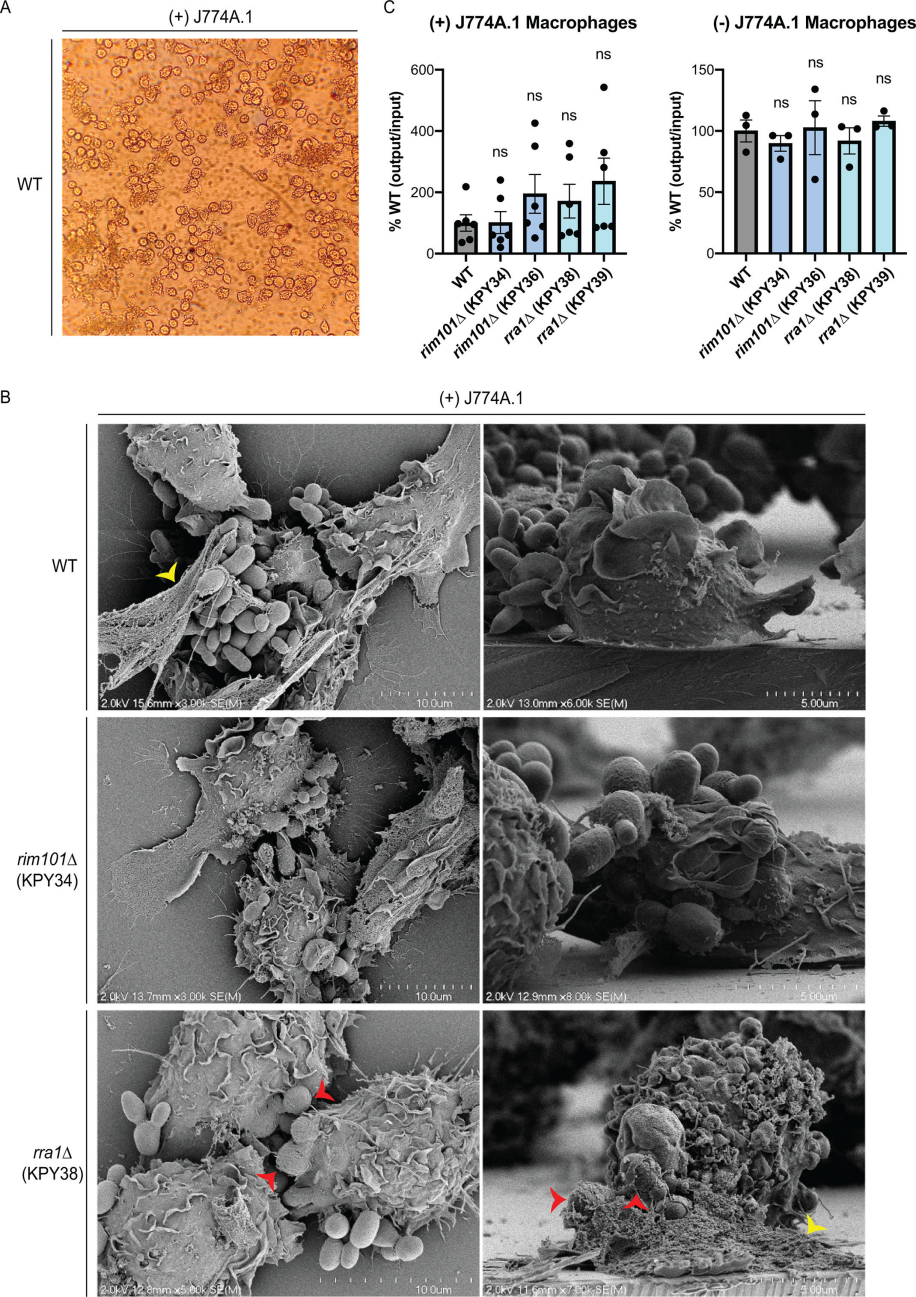
*M. sympodialis* interactions with macrophages. (**A**) Light microscopy image of the WT strain co-cultured with J774A.1 macrophage after 1 hour of co-culture. (**B**) The WT strain, *rim101*Δ mutant strains, and *rra1*Δ mutant strains were co-incubated with (left) and without (right) J774A.1 murine macrophages for 4 hours. Survival of the indicated fungal strains was assessed by quantitative culture, and the percentage of recovered (output) colony forming units (CFUs) compared to the original (input) CFUs was normalized to the WT strain [% WT (output/input)]. This experiment was performed with a minimum of three biological replicates (*n*  =  3). Error bars represent the SEM compared to WT. Log transformation was used to normally distribute the data for statistical analysis (one-way ANOVA; ns, not significant). (**C**) SEM images of the WT strain, the *rim101*Δ mutant strain (KPY34), and the *rra1*Δ mutant strain (KPY38) after 1 hour of co-culture with J774A.1 macrophage. Cells were fixed with 2.5% glutaraldehyde, dehydrated, critical point dried, sputter-coated with gold-palladium, and imaged. Red arrowheads indicate fungal cells actively undergoing macrophage phagocytosis. Yellow arrowheads indicate potential METs.

Due to its prevalence on the skin, *Malassezia* species have been studied for interactions with epidermis-resident keratinocytes, dendritic cells, and macrophages (24) as well as with the murine macrophage-like cell line J774A.1 *in vitro* (33). To assess the role of *M. sympodialis* Rim signaling in the interaction of fungal and innate immune cells, we performed a similar *in vitro* co-culture of the WT and Rim pathway mutants with J774A.1 cells. As early as 1 hour after co-culture, we observed macrophages clustering with fungal cells (Fig. 3A). We used SEM to further visualize the details of these physical interactions. We found that all tested strains [WT, *rim101*Δ (KPY34), and *rra1*Δ (KPY38)] were actively phagocytosed within 1 hour of co-culture (Fig. 3B). Typically, we observed groups of ~2–3 macrophages assembling to engulf clumps of fungal cells. In some cases, the macrophage/fungal cell association contained extracellular material, possibly consistent with macrophage extracellular traps (METs) (46) (Fig. 3B, yellow arrowheads).

To assess the ability of this macrophage-like cell line to rapidly kill the fungal cells, we also tested for survival differences among the three *M. sympodialis* strains in this co-culture system (33). We chose a short co-incubation period to address pH-related growth effects in the *rim101*Δ and *rra1*Δ strains. After 4 hours of co-culture with J774A.1 cells**,** we observed no significant survival differences between the WT, *rim101*Δ, and *rra1*Δ mutants (Fig. 3C, left). Despite the alkaline pH growth defect of the two mutants, there was no loss of viability of either mutant strain when incubated in a tissue culture medium without macrophages during this short period of incubation (Fig. 3C, right). Collectively, these observations indicate that macrophages recognize and actively phagocytose *Ms*. However, this association does not result in rapid fungal cell killing.

### *M. sympodialis* elicits a robust TNF response when co-cultured with macrophages independent of Rim pathway signaling

**Fig 4:**
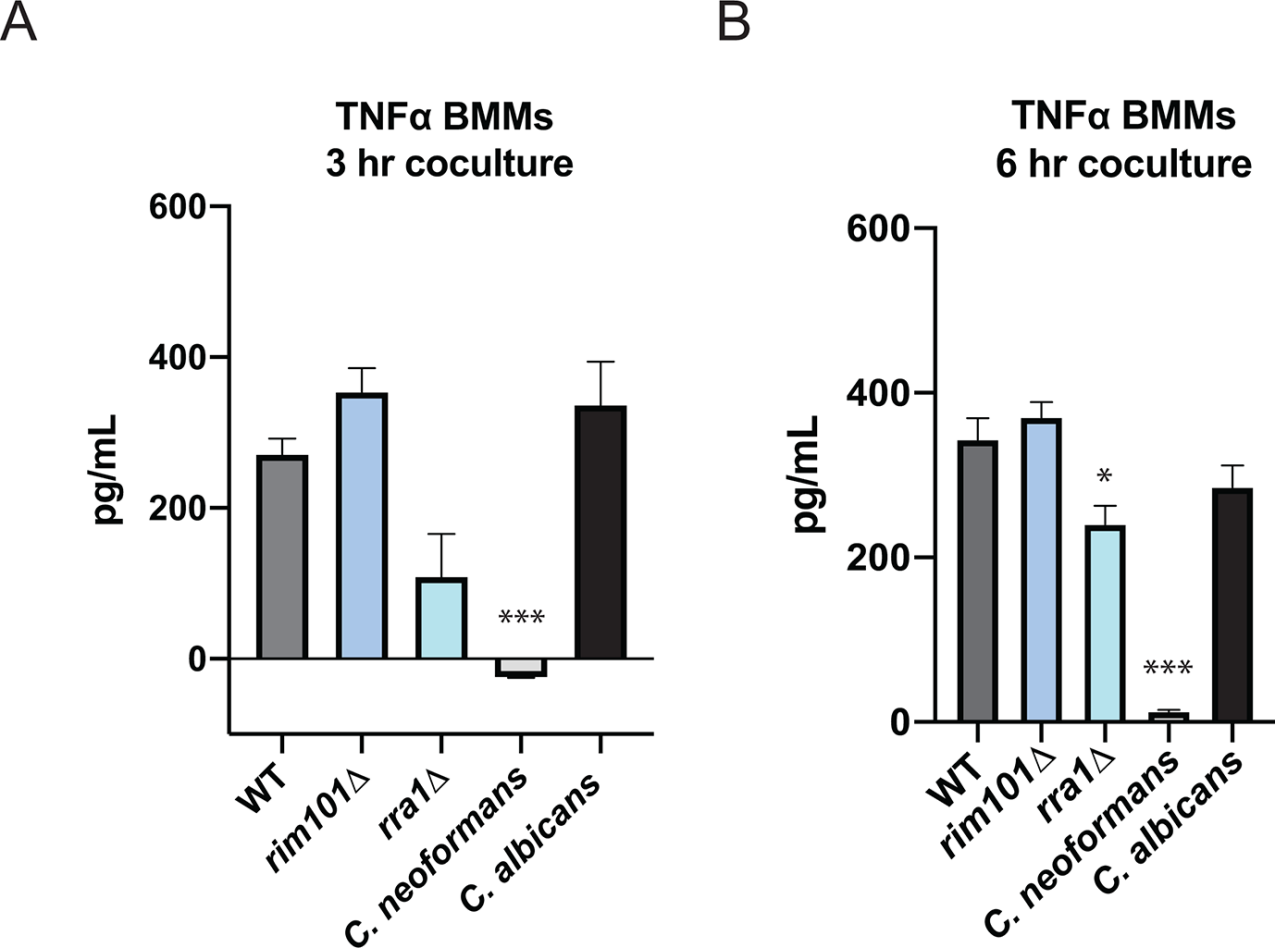
*M. sympodialis* elicits TNF response when co-cultured with macrophages. The *M. sympodialis* WT, *rim101*Δ, and *rra1*Δ strains were incubated for 3 days in DMEM +10% FBS at 30°C preceding a final incubation at 37°C for 16 hours prior to co-culture. WT *C. neoformans* (strain H99) cells were incubated for 2 days in DMEM +10% FBS at 30°C preceding a final incubation at 37°C for 16 hours prior to co-culture. WT *C. albicans* (SC5314) cells were incubated for 1 day in DMEM without serum at 30°C prior to co-culture. BMMs were co-incubated with the indicated fungal strains for 3 hours (**A**) or 6 hours (**B**) at a multiplicity of infection of 10:1, fungal cells:BMMs. TNF levels (pg/mL) were assayed from the co-culture supernatant by ELISA. Data represent means from six replicates per strain per condition. One-way ANOVA and Tukey’s multiple comparison test were used to compare means. ****P < 0.0001; ***P < 0.001; *P = 0.0186. Statistical comparisons were made against WT *M. sympodialis*.

Prior investigations into the ability of *Malassezia* to activate macrophages have yielded conflicting results, with individual studies suggesting that *Malassezia* species may either trigger or inhibit macrophage activation. These discrepancies have been attributed to differences in the growth phase of the cells, the fungal species assayed, and the nature of the protective lipid layer surrounding *Malassezia* cells (47–49). We quantified the production of TNF following the co-incubation of primary BMMs with the *M. sympodialis* wildtype, *rim101*∆ mutant, and *rra1*∆ mutant strains. We also measured TNF production in response to co-culture with wild-type *Candida albicans* and *Cryptococcus neoformans* strains, to serve as positive and negative controls, respectively. Following both a 3-hour and 6-hour co-culture (Fig. 4A and B), we observed an expected profound induction of TNF by the macrophages co-cultured with *C. albicans* cells (50). We also noted undetectable levels of TNF after co-culture with wild-type *C. neoformans* cells, consistent with prior studies documenting an effective immune evasion phenotype by wild-type *C. neoformans* cells (17). Similar to the case with *C. albicans*, BMMs exposed to all three *M. sympodialis* strains produced high levels of TNF (Fig. 4A and B). To determine whether this level of TNF by BMMs required viable *M. sympodialis* cells, we repeated the assay with heat-killed fungal cells. We observed similar levels of TNF production as with live cells in all strains tested (Fig. S2), suggesting that the TNF production is most likely primarily due to physical interaction between the macrophage and structural features of the fungal cell, as observed in other fungal species (34). Exposure of the BMMs to the *Ms rra1*Δ mutant strain resulted in variably lower levels of TNF stimulation compared to the WT and *rim101*Δ mutant, though not resulting in the complete suppression of macrophage activation as observed with the *C. neoformans* control (Fig. S2) (17). Together, these data suggest that the cell changes associated with mutation of the *M. sympodialis RIM101* gene do not affect TNF production by co-cultured macrophages, and a *Ms rra1*Δ mutation has only a partial effect. Moreover, the degree of macrophage TNF production in response to *Ms* is similar to that observed in co-cultures with *C. albicans*. By contrast, *M. sympodialis* does not share the immune avoidance phenotype of *C. neoformans* in this assay.

### The Rim/Pal pathway impacts *M. sympodialis* fitness in a murine model of atopic dermatitis

**Fig 5:**
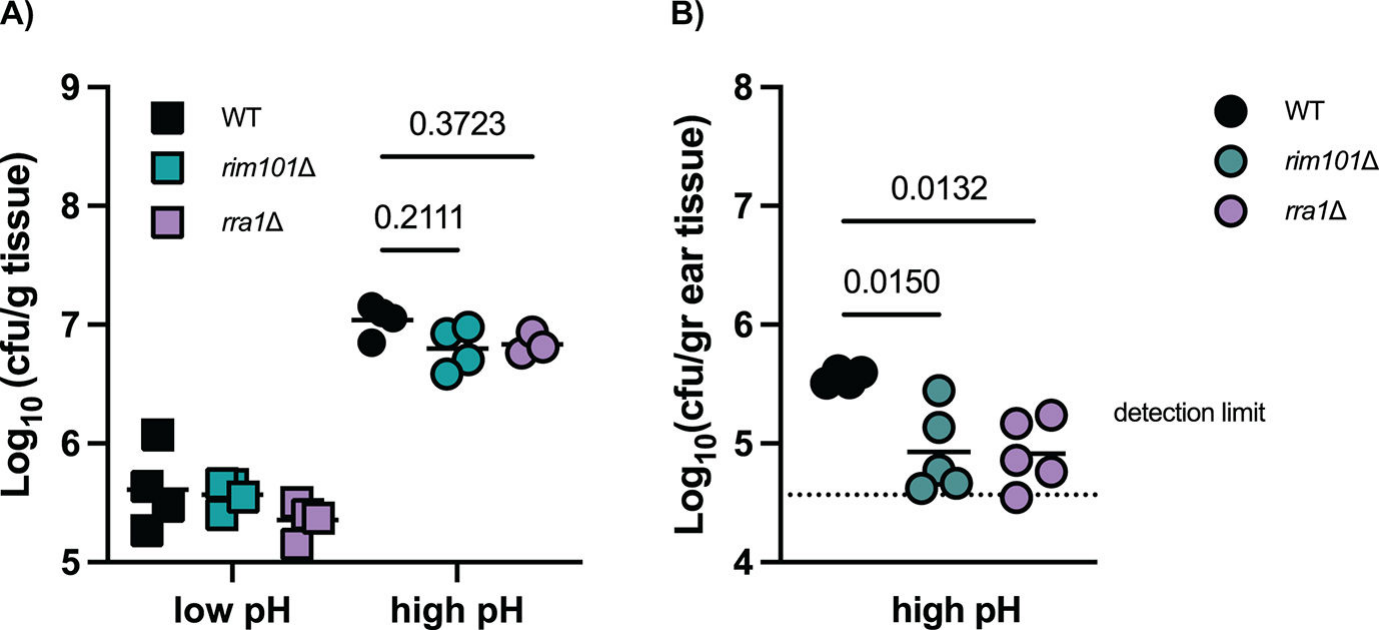
The Rim/Pal1 pathway impacts *M. sympodialis* fitness in a murine model of atopic dermatitis. The ear skin of WT C57BL/6 mice was repeatedly treated with either an ethanol solvent control (low pH, panel A) or with MC903 (high pH, panels A and B) for 10 days to induce an atopic dermatitis-like state. Treated skin was then associated dorsally with *M. sympodialis* WT, *rim101*∆ (KPY34), and *rra1*∆ (KPY36). The skin fungal load was quantified after 4 days (**A**)or 7 days (**B**)of colonization. Each symbol represents one animal. The mean of each group is indicated (dotted line, detection limit). Two-way ANOVA (**A**)or one-way ANOVA (**B**)were used to determine the statistical significance of the mean colony-forming units (CFU) of each mutant against WT *M. sympodialis*. *P < 0.05.

Finally, we assessed the impact of the Rim/Pal pathway on the interaction of *M. sympodialis* with the host skin *in vivo* under high pH conditions reminiscent of those in the skin of atopic dermatitis patients (39). Atopy-like conditions were induced by repeated administration of the vitamin D analog MC903 to the murine skin prior to the fungal association (39). WT *M. sympodialis* robustly colonized the murine skin by day 4 post-infection, with higher loads in the atopic dermatitis-like skin than in control skin, as previously shown (38) (Fig. 5A). Importantly, under high pH, but not low pH conditions, colonization levels of both, the *rim101*∆ [KPY34] and *rra1*∆ [KPY36] mutants were reduced compared to those of the WT control strain, although differences did not reach statistical significance (Fig. 5A). The effect was more pronounced at day 7 when the lack of *RIM101* and *RRA1* clearly impaired fitness and resistance to clearance of *M. sympodialis* in the skin with elevated pH (Fig. 5B). Overall reduced fungal burden on day 7 vs. day 4 is consistent with the previous observation that *Malassezia* skin colonization is transient in experimental mice (24, 38). The diminished capacity of the *rim101*∆ and *rra1*∆ mutants to colonize the atopic dermatitis-like murine skin was not due to an intrinsic growth defect of these strains or a defect to initially establish cutaneous colonization, as they colonized the skin equally well at low pH (Fig. 5A). The difference in skin colonization levels between WT, *rim101,* and *rra1* mutant strains did not impact the degree of inflammation of the atopic dermatitis-like skin, as assessed by quantification of ear swelling (Fig. S2A and B). Together, these data demonstrate the importance of *Ms* Rim signaling in fungal survival at alkaline pH, both *in vitro* as well as in physiologically relevant sites for this commensal microorganism.

## DISCUSSION

*Malassezia* species are among the most common commensal fungi present on the skin, and they are increasingly noted as frequent components of the human gut microbiome (51). Although most skin sites have a relatively acidic pH, the pH of skin can vary dramatically based on health and disease states. Similar to other fungi, *Malassezia* species possess genes predicted to encode the major components of the pH-responsive Rim signal transduction pathway. We have shown that an intact Rim pathway is required for survival on neutral to alkaline pH as well as elevated salt concentrations, similar to other fungal species. We have also demonstrated that the *M. sympodialis* Rra1 protein is required for fungal survival at alkaline pH, suggesting that Rra1 orthologs are conserved pH-responsive upstream components of Rim signaling in basidiomycetes, likely serving a similar function as the ascomycete Rim21 pH sensors (18). Finally, we provide evidence that the Rim101/Rra1 pathway increases the fitness of *Malassezia in vivo* in skin exhibiting an elevated pH as is the case in atopic dermatitis.

Our RNA-Seq analysis of the *M. sympodialis rim101*Δ and *rra1*Δ mutants demonstrated strikingly similar patterns of transcription, further functionally linking *Ms*Rim101 and *Ms*Rra1. Genes demonstrating similar patterns of transcriptional regulation by both Rim101 and Rra1 include those encoding membrane transporters and proteins involved in cell surface adaptation and intracellular trafficking. Proteins involved in similar cell processes are regulated by the Rim101/PacC protein in other fungal species, including the related basidiomycete *C. neoformans* (52). The fungal cell wall undergoes dramatic changes in structure in response to increases in pH (17), resulting in enhanced fungal survival in this new environmental condition. Therefore, defects in Rim signaling, and the associated failure of pH-responsive cell wall adaptations, may be a major reason for the alkaline pH growth sensitivity in Rim pathway mutant strains.

We also observed Rim pathway-dependent transcriptional changes in genes involved in membrane lipid biosynthesis. This observation is consistent with recent studies in *C. neoformans* Rim signaling in which the phospholipid asymmetry and composition of membranes affect Rim pathway activation (52). More detailed analysis of the *M. sympodialis* transcriptome is limited by the incomplete annotation of the recently assembled *M. sympodialis* genome (25), with many of the genes demonstrating Rim pathway-dependent expression being listed as uncharacterized proteins. However, these data further demonstrate how the fungal-specific Rim/Pal signaling cascade directs adaptive cellular changes to address the unique challenges resulting from alkaline extracellular environments. They also suggest that basidiomycetes and ascomycetes have incorporated structurally related but distinct proteins as pH sensors at the plasma membrane.

Interestingly, Rim101 regulation only accounts for a subset of the genes that are upregulated or downregulated in response to alkaline pH. However, a large portion of the genes whose expression appears to be Rim101-dependent overlap with the upregulated or downregulated genes in the WT in response to DMEM. Overall, these data suggest that many Rim101-regulated processes are conserved between *M. sympodialis* and other distantly related fungal species.

In many fungal species, mutations in Rim/Pal pathway signaling elements result in a marked attenuation of virulence. For example, *C. albicans* Rim pathway mutants are defective in the yeast-hyphal transition in response to elevation in pH, and they are accordingly avirulent in animal models of infection (53). Related mutations in the *Aspergillus fumigatus* Pal/PacC pathway display reduced hyphal growth in infected lungs (42). In the case of *C. neoformans*, Rim pathway mutants are defective in many phenotypes typically associated with pathogenesis in this species: these mutant strains are unable to grow well at mammalian pH, as well as in the presence of iron deprivation. Moreover, *C. neoformans rim* mutants fail to incorporate capsular polysaccharides on the cell surface (10). Consistent with these *in vitro* observations, *C. neoformans rim* mutants have reduced fungal burdens as assessed by quantitative cultures of infected lungs in animal models of cryptococcosis (10). Paradoxically, mice infected with these attenuated *rim* mutant strains display decreased survival compared to mice infected with wild-type strains (10, 18). This observation has been explained by excessive immunopathology in the *rim* mutant infections. *C. neoformans* Rim pathway mutations result in unmasking of typically hidden and immunogenic cell wall epitopes, leading to hyperstimulation of innate immune cell activation and accelerated tissue damage (18).

We did not observe Rim pathway-dependent changes in the degree to which *M. sympodialis* activates macrophages *in vitro*. The *Ms* wildtype, *rim101*Δ, and *rra1*Δ strains induced similar levels of TNF production during *in vitro* macrophage/fungal co-culture experiments. However, in contrast to *C. neoformans* in which the wild-type strains effectively suppress macrophage activation, *M. sympodialis* strains display constitutively high levels of macrophage TNF production, similar to *C. albicans*. Therefore, *M. sympodialis*, though a common commensal, does not appear to shield itself from immune recognition as efficiently as encapsulated fungi such as *C. neoformans*. To fully understand the role of the Rim/Pal1 pathway in the antifungal response to *Malassezia*, future studies with skin-resident cells and model systems harboring features characteristic of the cutaneous niche will be needed.

Emerging data from microbiome studies suggest that *Malassezia* species are not only common skin colonizers but are also found in the human gut, where the pH varies widely from very acidic in the stomach to more alkaline in the small and large intestines. The presence of *Malassezia* species in these micro-niches has been associated with longer-term sequelae of continuous antigenic stimulation, including inflammatory bowel disease (54). Further exploration of the interaction of this commensal fungus with the immune system may elucidate ways in which *Malassezia* species might contribute to health or disease at specific anatomic sites.

## Data Availability

Processed RNA-seq seq data are available in the supplemental Tables; raw and processed data are available through NCBI Gene Expression Omnibus (GEO) accession number GSE254653.

## SUPPLEMENTAL MATERIAL

Figure S1 mbio.02060-24-s0001.eps Correlation of gene expression in the *rim101* and *rra1* strains.

Figure S2 mbio.02060-24-s0002.eps *M. sympodialis* TNF induction is not dependent on fungal cell viability.

Figure S3 mbio.02060-24-s0003.eps The *M. sympodialis* Rim101/Rra1 pathway does not impact inflammation in a murine model of atopic dermatitis.

Supplemental material mbio.02060-24-s0004.docx Tables S1-S4.

Tables S5-S20 mbio.02060-24-s0005.xlsx Comparative transcriptional analysis of WT, *rim101*, and *rra1* strains.

Legends mbio.02060-24-s0006.docx Supplemental figure legends.

ASM does not own the copyrights to Supplemental Material that may be linked to, or accessed through, an article. The authors have granted ASM a non-exclusive, world-wide license to publish the Supplemental Material files. Please contact the corresponding author directly for reuse.

## References

[1] Ali SM, Yosipovitch G. 2013. Skin pH: from basic science to basic skin care. Acta Derm Venereol 93:261–267. doi:10.2340/00015555-1531 23322028

[2] Selander C, Zargari A, Möllby R, Rasool O, Scheynius A. 2006. Higher pH level, corresponding to that on the skin of patients with atopic eczema, stimulates the release of Malassezia sympodialis allergens. Allergy 61:1002–1008. doi:10.1111/j.1398-9995.2006.01108.x 16867055

[3] Aykut B, Pushalkar S, Chen R, Li Q, Abengozar R, Kim JI, Shadaloey SA, Wu D, Preiss P, Verma N, Guo Y, Saxena A, Vardhan M, Diskin B, Wang W, Leinwand J, Kurz E, Kochen Rossi JA, Hundeyin M, Zambrinis C, Li X, Saxena D, Miller G. 2019. The fungal mycobiome promotes pancreatic oncogenesis via activation of MBL. Nature 574:264–267. doi:10.1038/s41586-019-1608-2 31578522 PMC6858566

[4] Selvig K, Alspaugh JA. 2011. pH response pathways in fungi: adapting to host-derived and environmental signals. Mycobiology 39:249–256. doi:10.5941/MYCO.2011.39.4.249 22783112 PMC3385132

[5] Cornet M, Gaillardin C. 2014. pH signaling in human fungal pathogens: a new target for antifungal strategies. Eukaryot Cell 13:342–352. doi:10.1128/EC.00313-13 24442891 PMC3957587

[6] Peñalva MA, Tilburn J, Bignell E, Arst HN Jr. 2008. Ambient pH gene regulation in fungi: making connections. Trends Microbiol 16:291–300. doi:10.1016/j.tim.2008.03.006 18457952

[7] Obara K, Yamamoto H, Kihara A. 2012. Membrane protein Rim21 plays a central role in sensing ambient pH in Saccharomyces cerevisiae. J Biol Chem 287:38473–38481. doi:10.1074/jbc.M112.394205 23019326 PMC3493892

[8] Arst HN, Bignell E, Tilburn J. 1994. Two new genes involved in signalling ambient pH in Aspergillus nidulans. Mol Gen Genet 245:787–790. doi:10.1007/BF00297286 7830727

[9] Tréton B, Blanchin-Roland S, Lambert M, Lépingle A, Gaillardin C. 2000. Ambient pH signalling in ascomycetous yeasts involves homologues of the Aspergillus nidulans genes palF and paIH. Mol Gen Genet 263:505–513. doi:10.1007/s004380051195 10821185

[10] O’Meara TR, Norton D, Price MS, Hay C, Clements MF, Nichols CB, Alspaugh JA. 2010. Interaction of Cryptococcus neoformans Rim101 and protein kinase A regulates capsule. PLoS Pathog. 6:e1000776. doi:10.1371/journal.ppat.1000776 20174553 PMC2824755

[11] Li W, Mitchell AP. 1997. Proteolytic activation of Rim1p, a positive regulator of yeast sporulation and invasive growth. Genetics 145:63–73. doi:10.1093/genetics/145.1.63 9017390 PMC1207785

[12] Díez E, Alvaro J, Espeso EA, Rainbow L, Suárez T, Tilburn J, Arst HN, Peñalva MA. 2002. Activation of the Aspergillus PacC zinc finger transcription factor requires two proteolytic steps. EMBO J 21:1350–1359. doi:10.1093/emboj/21.6.1350 11889040 PMC125927

[13] Tilburn J, Sarkar S, Widdick DA, Espeso EA, Orejas M, Mungroo J, Peñalva MA, Arst HN. 1995. The Aspergillus PacC zinc finger transcription factor mediates regulation of both acid- and alkaline-expressed genes by ambient pH. EMBO J 14:779–790. doi:10.1002/j.1460-2075.1995.tb07056.x 7882981 PMC398143

[14] O’Meara TR, Xu W, Selvig KM, O’Meara MJ, Mitchell AP, Alspaugh JA. 2014. The Cryptococcus neoformans Rim101 transcription factor directly regulates genes required for adaptation to the host. Mol Cell Biol 34:673–684. doi:10.1128/MCB.01359-13 24324006 PMC3911494

[15] Cornet M, Bidard F, Schwarz P, Da Costa G, Blanchin-Roland S, Dromer F, Gaillardin C. 2005. Deletions of endocytic components VPS28 and VPS32 affect growth at alkaline pH and virulence through both RIM101-dependent and RIM101-independent pathways in Candida albicans. Infect Immun 73:7977–7987. doi:10.1128/IAI.73.12.7977-7987.2005 16299290 PMC1307034

[16] Garnaud C, García-Oliver E, Wang Y, Maubon D, Bailly S, Despinasse Q, Champleboux M, Govin J, Cornet M. 2018. The rim pathway mediates antifungal tolerance in Candida albicans through newly identified Rim101 transcriptional targets, including Hsp90 and Ipt1. Antimicrob Agents Chemother 62:e01785-17. doi:10.1128/AAC.01785-17 29311085 PMC5826160

[17] O’Meara TR, Holmer SM, Selvig K, Dietrich F, Alspaugh JA. 2013. Cryptococcus neoformans Rim101 is associated with cell wall remodeling and evasion of the host immune responses. mBio 4:e00522-12. doi:10.1128/mBio.00522-12 23322637 PMC3551547

[18] Ost KS, O’Meara TR, Huda N, Esher SK, Alspaugh JA. 2015. The Cryptococcus neoformans alkaline response pathway: identification of a novel rim pathway activator. PLoS Genet 11:e1005159. doi:10.1371/journal.pgen.1005159 25859664 PMC4393102

[19] Cervantes-Chávez JA, Ortiz-Castellanos L, Tejeda-Sartorius M, Gold S, Ruiz-Herrera J. 2010. Functional analysis of the pH responsive pathway Pal/Rim in the phytopathogenic basidiomycete Ustilago maydis. Fungal Genet Biol 47:446–457. doi:10.1016/j.fgb.2010.02.004 20153837

[20] Aréchiga-Carvajal ET, Ruiz-Herrera J. 2005. The RIM101/pacC homologue from the basidiomycete Ustilago maydis is functional in multiple pH-sensitive phenomena. Eukaryot Cell 4:999–1008. doi:10.1128/EC.4.6.999-1008.2005 15947192 PMC1151993

[21] Pianalto KM, Ost KS, Brown HE, Alspaugh JA. 2018. Characterization of additional components of the environmental pH-sensing complex in the pathogenic fungus Cryptococcus neoformans. J Biol Chem 293:9995–10008. doi:10.1074/jbc.RA118.002741 29769315 PMC6028953

[22] Wu G, Zhao H, Li C, Rajapakse MP, Wong WC, Xu J, Saunders CW, Reeder NL, Reilman RA, Scheynius A, Sun S, Billmyre BR, Li W, Averette AF, Mieczkowski P, Heitman J, Theelen B, Schröder MS, De Sessions PF, Butler G, Maurer-Stroh S, Boekhout T, Nagarajan N, Dawson TL Jr. 2015. Genus-wide comparative genomics of Malassezia delineates its phylogeny, physiology, and niche adaptation on human skin. PLoS Genet 11:e1005614. doi:10.1371/journal.pgen.1005614 26539826 PMC4634964

[23] Ianiri G, Applen Clancey S, Lee SC, Heitman J. 2017. FKBP12-dependent inhibition of calcineurin mediates immunosuppressive antifungal drug action in Malassezia. mBio 8:e01752-17. doi:10.1128/mBio.01752-17 29066552 PMC5654937

[24] Sparber F, De Gregorio C, Steckholzer S, Ferreira FM, Dolowschiak T, Ruchti F, Kirchner FR, Mertens S, Prinz I, Joller N, Buch T, Glatz M, Sallusto F, LeibundGut-Landmann S. 2019. The skin commensal yeast Malassezia triggers a type 17 response that coordinates anti-fungal immunity and exacerbates skin inflammation. Cell Host Microbe 25:389–403. doi:10.1016/j.chom.2019.02.002 30870621

[25] Zhu Y, Engström PG, Tellgren-Roth C, Baudo CD, Kennell JC, Sun S, Billmyre RB, Schröder MS, Andersson A, Holm T, Sigurgeirsson B, Wu G, Sankaranarayanan SR, Siddharthan R, Sanyal K, Lundeberg J, Nystedt B, Boekhout T, Dawson TL Jr, Heitman J, Scheynius A, Lehtiö J. 2017. Proteogenomics produces comprehensive and highly accurate protein-coding gene annotation in a complete genome assembly of Malassezia sympodialis. Nucleic Acids Res. 45:2629–2643. doi:10.1093/nar/gkx006 28100699 PMC5389616

[26] Ianiri G, Boyce KJ, Idnurm A. 2017. Isolation of conditional mutations in genes essential for viability of Cryptococcus neoformans. Curr Genet 63:519–530. doi:10.1007/s00294-016-0659-2 27783209 PMC5405016

[27] Ianiri G, Averette AF, Kingsbury JM, Heitman J, Idnurm A. 2016. Gene function analysis in the ubiquitous human commensal and pathogen Malassezia genus. mBio 7:e01853-16. doi:10.1128/mBio.01853-16 27899504 PMC5137500

[28] Dobin A, Davis CA, Schlesinger F, Drenkow J, Zaleski C, Jha S, Batut P, Chaisson M, Gingeras TR. 2013. STAR: ultrafast universal RNA-seq aligner. Bioinformatics 29:15–21. doi:10.1093/bioinformatics/bts635 23104886 PMC3530905

[29] Love MI, Anders S, Kim V, Huber W. 2015. RNA-Seq workflow: gene-level exploratory analysis and differential expression. F1000Res 4:1070. doi:10.12688/f1000research.7035.1 26674615 PMC4670015

[30] Love MI, Huber W, Anders S. 2014. Moderated estimation of fold change and dispersion for RNA-seq data with DESeq2. Genome Biol 15:550. doi:10.1186/s13059-014-0550-8 25516281 PMC4302049

[31] Alvarez-Jarreta J, Amos B, Aurrecoechea C, Bah S, Barba M, Barreto A, Basenko EY, Belnap R, Blevins A, Böhme U, et al.. 2024. VEuPathDB: the eukaryotic pathogen, vector and host bioinformatics resource center in 2023. Nucleic Acids Res. 52:D808–D816. doi:10.1093/nar/gkad1003 37953350 PMC10767879

[32] Esher SK, Ost KS, Kozubowski L, Yang D-H, Kim MS, Bahn Y-S, Alspaugh JA, Nichols CB. 2016. Relative contributions of prenylation and postprenylation processing in Cryptococcus neoformans pathogenesis. mSphere 1:e00084-15. doi:10.1128/mSphere.00084-15 27303728 PMC4894686

[33] Ianiri G, Coelho MA, Ruchti F, Sparber F, McMahon TJ, Fu C, Bolejack M, Donovan O, Smutney H, Myler P, Dietrich F, Fox D 3rd, LeibundGut-Landmann S, Heitman J. 2020. HGT in the human and skin commensal Malassezia: a bacterially derived flavohemoglobin is required for NO resistance and host interaction. Proc Natl Acad Sci U S A 117:15884–15894. doi:10.1073/pnas.2003473117 32576698 PMC7354939

[34] Ost KS, Esher SK, Leopold Wager CM, Walker L, Wagener J, Munro C, Wormley FL Jr, Alspaugh JA. 2017. Rim pathway-mediated alterations in the fungal cell wall influence immune recognition and inflammation. mBio 8:e02290-16. doi:10.1128/mBio.02290-16 28143983 PMC5285508

[35] Esher SK, Ost KS, Kohlbrenner MA, Pianalto KM, Telzrow CL, Campuzano A, Nichols CB, Munro C, Wormley FL, Alspaugh JA. 2018. Defects in intracellular trafficking of fungal cell wall synthases lead to aberrant host immune recognition. PLoS Pathog 14:e1007126. doi:10.1371/journal.ppat.1007126 29864141 PMC6002136

[36] Russell WMS. 1995. The development of the three Rs concept. Altern Lab Anim 23:298–304. doi:10.1177/026119299502300 11656565

[37] Moosbrugger-Martinz V, Schmuth M, Dubrac S. 2017. A mouse model for atopic dermatitis using topical application of vitamin D3 or of its analog MC903. Methods Mol Biol 1559:91–106. doi:10.1007/978-1-4939-6786-5_8 28063040

[38] Ruchti F, Zwicky P, Becher B, Dubrac S, LeibundGut-Landmann S. 2024. Epidermal barrier impairment predisposes for excessive growth of the allergy-associated yeast Malassezia on murine skin. Allergy 79:1531–1547. doi:10.1111/all.16062 38385963

[39] Xie L, McKenzie CI, Qu X, Mu Y, Wang Q, Bing N, Naidoo K, Alam MJ, Yu D, Gong F, Ang C, Robert R, Marques FZ, Furlotte N, Hinds D, Gasser O, 23andMe Research Team, Xavier RJ, Mackay CR. 2021. pH and proton sensor GPR65 determine susceptibility to atopic dermatitis. J Immunol 207:101–109. doi:10.4049/jimmunol.2001363 34135065 PMC8674371

[40] Sparber F, LeibundGut-Landmann S. 2019. Infecting mice with Malassezia spp. to study the fungus-host interaction. J Vis Exp 153:31762452. doi:10.3791/60175 31762452

[41] Bignell E, Negrete-Urtasun S, Calcagno AM, Haynes K, Arst HN, Rogers T. 2005. The Aspergillus pH-responsive transcription factor PacC regulates virulence. Mol Microbiol 55:1072–1084. doi:10.1111/j.1365-2958.2004.04472.x 15686555

[42] Bertuzzi M, Schrettl M, Alcazar-Fuoli L, Cairns TC, Muñoz A, Walker LA, Herbst S, Safari M, Cheverton AM, Chen D, Liu H, Saijo S, Fedorova ND, Armstrong-James D, Munro CA, Read ND, Filler SG, Espeso EA, Nierman WC, Haas H, Bignell EM. 2014. The pH-responsive PacC transcription factor of Aspergillus fumigatus governs epithelial entry and tissue invasion during pulmonary aspergillosis. PLoS Pathog. 10:e1004413. doi:10.1371/journal.ppat.1004413 25329394 PMC4199764

[43] Porta A, Ramon AM, Fonzi WA. 1999. PRR1, a homolog of Aspergillus nidulans palF, controls pH-dependent gene expression and filamentation in Candida albicans. J Bacteriol 181:7516–7523. doi:10.1128/JB.181.24.7516-7523.1999 10601209 PMC94209

[44] Ramon AM, Porta A, Fonzi WA. 1999. Effect of environmental pH on morphological development of Candida albicans is mediated via the PacC-related transcription factor encoded by PRR2. J Bacteriol 181:7524–7530. doi:10.1128/JB.181.24.7524-7530.1999 10601210 PMC94210

[45] Brown HE, Telzrow CL, Saelens JW, Fernandes L, Alspaugh JA. 2020. Sterol-response pathways mediate alkaline survival in diverse fungi. mBio 11:e00719-20. doi:10.1128/mBio.00719-20 32546619 PMC7298709

[46] Doster RS, Rogers LM, Gaddy JA, Aronoff DM. 2018. Macrophage extracellular traps: a scoping review. J Innate Immun 10:3–13. doi:10.1159/000480373 28988241 PMC6757166

[47] Kesavan S, Walters CE, Holland KT, Ingham E. 1998. The effects of Malassezia on pro-inflammatory cytokine production by human peripheral blood mononuclear cells in vitro. Med Mycol 36:97–106. doi:10.1080/02681219880000161 9776820

[48] Kesavan S, Holland KT, Ingham E. 2000. The effects of lipid extraction on the immunomodulatory activity of Malassezia species in vitro. Med Mycol 38:239–247. doi:10.1080/mmy.38.3.239.247 10892993

[49] Ashbee HR, Evans EGV. 2002. Immunology of diseases associated with Malassezia species. Clin Microbiol Rev 15:21–57. doi:10.1128/CMR.15.1.21-57.2002 11781265 PMC118058

[50] Jeremias J, Kalo-Klein A, Witkin SS. 1991. Individual differences in tumour necrosis factor and interleukin-1 production induced by viable and heat-killed Candida albicans. J Med Vet Mycol 29:157–163. doi:10.1080/02681219180000261 1890562

[51] Scheynius A, Johansson C, Buentke E, Zargari A, Linder MT. 2002. Atopic eczema/dermatitis syndrome and Malassezia. Int Arch Allergy Immunol 127:161–169. doi:10.1159/000053860 11979041

[52] Brown HE, Ost KS, Esher SK, Pianalto KM, Saelens JW, Guan Z, Andrew Alspaugh J. 2018. Identifying a novel connection between the fungal plasma membrane and pH-sensing. Mol Microbiol 109:474–493. doi:10.1111/mmi.13998 29885030 PMC6173979

[53] Davis D. 2003. Adaptation to environmental pH in Candida albicans and its relation to pathogenesis. Curr Genet 44:1–7. doi:10.1007/s00294-003-0415-2 12819929

[54] Limon JJ, Tang J, Li D, Wolf AJ, Michelsen KS, Funari V, Gargus M, Nguyen C, Sharma P, Maymi VI, Iliev ID, Skalski JH, Brown J, Landers C, Borneman J, Braun J, Targan SR, McGovern DPB, Underhill DM. 2019. Malassezia is associated with Crohn’s disease and exacerbates colitis in mouse models. Cell Host Microbe 25:377–388. doi:10.1016/j.chom.2019.01.007 30850233 PMC6417942

