# Supplementary material for: Malassezia responds to environmental pH signals through the conserved Rim/Pal pathway
Source: mBio. 2024 Aug 27;15(10):e02060-24. doi: 10.1128/mbio.02060-24 (PMC11481519; doi:10.1128/mbio.02060-24)
Supplement: Supplemental material — Tables S1-S4. [file mbio.02060-24-s0004.docx]

Supplemental Tables – Pianalto, *et al*.

**Table S1: Fungal strains**

| **Strain Name** | **Genotype** | **Source** |
| --- | --- | --- |
| ATCC 42132 | *M. sympodialis* | [27] |
| KPY34 | *M. sympodialis rim101*Δ - 1 | This study |
| KPY35 | *M. sympodialis rim101*Δ - 2 | This study |
| KPY36 | *M. sympodialis* *rra1*Δ - 1 | This study |
| KPY37 | *M. sympodialis* *rra1*Δ - 2 | This study |
| H99  SC5314 | *Cryptococcus neoformans* (WT)  *C. albicans* (WT) | [51]  [52] |

**Table S2: Gene deletion construct primers**

| **Primer Name** | **Sequence 5’ - 3’** | **Construct** |
| --- | --- | --- |
| *Deletion construct primers* | | |
| ALID2078 | TCCACGGTGCAGATCCTC | *Malassezia* NAT/NEO marker F |
| ALID2081 | CGTCCTCTCCTATGTCTG | *Malassezia* NAT/NEO marker R |
| AA5328 | GCGCGCCTAGGCCTCTGCAGGTCGACT  CTGCTGGCAGACGCACCTAACAC | *RIM101* KO 1 |
| AA5329 | GAGGATCTGCACCGTGGAGGGTGTTGG  TGTGGAAACACACC | *RIM101* KO 2 |
| AA5330 | CAGACATAGGAGAGGACGCCAACACGG  GTCACAAGTTGCC | *RIM101* KO 3 |
| AA5331 | TGATTACGAATTCTTAATTAAGATATCGA  GCTGCACGCGATGCAGTGTCTC | *RIM101* KO 4 |
| AA5332 | GCGCGCCTAGGCCTCTGCAGGTCGACTC  TGCGCACGAATTCTCGCTCGAGT | *RRA1* KO 1 |
| AA5333 | GAGGATCTGCACCGTGGACGCGGCCGC  CTATTTCGCT | *RRA1* KO 2 |
| AA5334 | CAGACATAGGAGAGGACGCCGTGCTCA  AGACGGTCATCA | *RRA1* KO 3 |
| AA5335 | TGATTACGAATTCTTAATTAAGATATCG  AGCAGCTGTCGCAGGTGCTGTG | *RRA1* KO 4 |
| JOHE43282 | CACCAGGGTTTCCAGTCTC | *NAT* marker F primer |
| JOHE43281 | GTCGGAGAAGCAGTCAATGC | *NAT* marker R primer |

**Table S3: Confirmation PCR primers**

| **Primer name** | **Sequence 5’-3’** | **Target** |
| --- | --- | --- |
| AA5340 | CGTCGTCGTGAGGGCAAGT | *Ms* *RIM101* KO F |
| AA5341 | CGATGCCCTGCTGGATCTTG | *Ms* *RIM101* KO R |
| AA5342 | GAAGGAGAAGCGTGCGCAG | *Ms* *RRA1* KO F |
| AA5343 | CCTCGTACCTGCGTCTCTGG | *Ms* *RRA1* KO R |

| **Primer name** | **Sequence 5’-3’** | **Gene target** |
| --- | --- | --- |
| JOHE45549 | TGCCGGAGCTCACCTCGCA | *TUB2* F |
| JOHE45550 | TACGACGAGTTCTTGGTCTG | *TUB2* R |
| AA5430 | TCCTCTAGCACCAAGCGATC | *RIM101* F |
| AA5431 | CACGTAACAATTGTGCAATGC | *RIM101* R |

**Table S4: Real-time PCR primers**
