# Supplementary material for: Malassezia responds to environmental pH signals through the conserved Rim/Pal pathway
Source: mBio. 2024 Aug 27;15(10):e02060-24. doi: 10.1128/mbio.02060-24 (PMC11481519; doi:10.1128/mbio.02060-24)
Supplement: Legends — Supplemental figure legends. [file mbio.02060-24-s0006.docx]

**Supplemental Figure Legends**

**Figure S1. Correlation of gene expression in the *rim101*Δ and *rra1*Δ strains.** The *Ms* WT, *rim101*Δ, and *rra1*Δ strains were incubated for 90 minutes in mDixon medium pH 4, mDixon medium pH 7.5, or DMEM tissue culture medium (pH 7.4). Deep RNA sequencing was performed for each strain at each condition, and the log_2_ fold-change for each gene was calculated for the *rim101*Δ and *rra1*Δ strains compared to the WT strain. Gene expression is highly correlated between *rim101*Δ and *rra1*Δ mutants at pH 7.5 and in DMEM, but not at pH 4. Red points indicate genes with expression in *rim101*Δ and *rra1*Δ that is significantly different from WT (adjusted p-value <= 0.05).

**Figure S2. *M. sympodialis* TNF induction is not dependent on fungal cell viability.** WT, *rim101*∆ (KPY34), and *rra1*∆ (KPY36) *M. sympodialis* mutant strains were incubated for 3 days in DMEM + 10% FBS and PenStrep at 30°C preceding a final incubation at 37°C for 16 hours prior to co-culture. WT (H99) *C. neoformans* cells were incubated for 2 days in DMEM + 10% FBS and PenStrep at 30°C preceding a final incubation at 37°C for 16 hours prior to co-culture. WT *C. albicans* (SC5314) cells were incubated for 1 day in DMEM without serum at 30°C prior to co-culture. Bone marrow-derived macrophages (BMMs) were co-incubated for 3 hours with heat-killed (HK) fungal cells (HK, 1 hour at 65°C). BMMs were co-incubated with fungal cells for 6 hours following a 3-hour LPS priming. All strains were at a multiplicity of infection (MOI) of 10:1, fungal cells : BMMs. TNF levels (pg/ml) were assayed from the co-culture supernatant by ELISA. Data represent means from 6 replicates per strain per condition. One-way ANOVA and Tukey’s multiple comparison test were used to compare means. ****, p < 0.0001; *, p < 0.006. Statistical comparisons were made against WT *M. sympodialis*.

**Figure S3. The *M. sympodialis* Rim101/Rra1 pathway does not impact inflammation in a murine model of atopic dermatitis**. To simulate high and low pH under *in vivo* conditions, the ear skin of WT C57BL/6 mice was treated with MC903 (high pH) or EtOH (solvent control, low pH) and then associated with *M. sympodialis* WT, *rim101*∆ [KPY34] and *rra1*∆ [KPY36]. The treatment schedule is indicated at the top of the graph with solid arrows indicating dorsal and ventral application of MC903 or EtOH, and dashed arrows indicate ventral application only of MC903 or EtOH. The ear thickness of mice was determined at the indicated time points. Each datapoint is the mean +/- SD of 4 (**A**) or 5 mice (**B**), respectively.
